# Variance of allele balance calculated from low coverage sequencing data infers departure from a diploid state

**DOI:** 10.1186/s12859-022-04685-z

**Published:** 2022-04-25

**Authors:** Kyle Fletcher, Rongkui Han, Diederik Smilde, Richard Michelmore

**Affiliations:** 1 The Genome Center, University of California, Davis, USA; 2 The Plant Biology Graduate Group, University of California, Davis, CA 95616 USA; 3 Naktuinbouw, Postbus 40, Sotaweg 22, 2370 AA Roelofarendsveen, The Netherlands; 4 Departments of Plant Sciences, Molecular and Cellular Biology, Medical Microbiology and Immunology, University of California, Davis, USA

**Keywords:** Heterokaryosis, Polyploidy, Oomycete, *Phytophthora*, *Bremia lactucae*, *Saccharomyces*, *Arabidopsis*, Allele frequency

## Abstract

**Background:**

Polyploidy and heterokaryosis are common and consequential genetic phenomena that increase the number of haplotypes in an organism and complicate whole-genome sequence analysis. Allele balance has been used to infer polyploidy and heterokaryosis in diverse organisms using read sets sequenced to greater than 50× whole-genome coverage. However, sequencing to adequate depth is costly if applied to multiple individuals or large genomes.

**Results:**

We developed VCFvariance.pl to utilize the variance of allele balance to infer polyploidy and/or heterokaryosis at low sequence coverage. This analysis requires as little as 10× whole-genome coverage and reduces the allele balance profile down to a single value, which can be used to determine if an individual has two or more haplotypes. This approach was validated using simulated, synthetic, and authentic read sets from the oomycete species *Bremia lactucae* and *Phytophthora infestans*, the fungal species *Saccharomyces cerevisiae*, and the plant species *Arabidopsis arenosa*. This approach was deployed to determine that nine of 21 genotyped European race-type isolates of *Bremia lactucae* were inconsistent with diploidy and therefore likely heterokaryotic.

**Conclusions:**

Variance of allele balance is a reliable metric to detect departures from a diploid state, including polyploidy, heterokaryosis, a mixed sample, or chromosomal copy number variation. Deploying this strategy is computationally inexpensive, can reduce the cost of sequencing by up to 80%, and used to test any organism.

**Supplementary Information:**

The online version contains supplementary material available at 10.1186/s12859-022-04685-z.

## Background

Polyploidy is a pervasive, well-known feature in all domains of eukaryotic life. Heterokaryosis is also common in eukaryotic microbes. Both are biologically consequential departures from diploidy. Polyploidy is rarer in animals, but rife in plants where it has been described as a predominant component of sympatric speciation [1, 2]. Increased cell size in plants is a consequence of polyploidy, possibly resulting in thicker, broader leaves and larger flowers, fruits, pollen, and stomata [1, 3]. Oomycetes (Stramenopiles), while somatically diploid, exhibit ploidy variation and/or heterokaryosis, which has consequences to fitness and virulence [4, 5]. Many fungal species are somatically haploid, but like oomycetes, exhibit ploidy variation and/or heterokaryosis [6–8]. Flow cytometry can be used to estimate nuclear DNA content and can directly infer polyploidy when multiple genome sizes are found within a species [9–11]. However, this may not be technically feasible for some species or situations and is ineffective in detecting heterokaryosis.

High-throughput, whole-genome sequencing has enabled the detection of polyploidy and heterokaryosis in multiple species [4, 5, 7, 8, 12–22]. Several in silico approaches have been developed to summarize nucleotide frequencies at polymorphic sites and determine if two or more haplotypes exist in a sample [5, 21]. These approaches resolve the number of haplotypes present in a DNA sample by inferring the allele balance at bi-allelic single nucleotide polymorphisms (SNPs). A 0.5/0.5 balance indicates two haplotypes (likely diploid), 0.33/0.67 indicates three haplotypes (likely triploid), and 0.25/0.5/0.75 indicates four haplotypes (likely tetraploid). This approach was used to determine the ploidy of isolates of *Phytophthora infestans* [12, 13] and has since been deployed in plants, fungi, and animals as well as other oomycetes [4, 8, 15–22]. In the case of the oomycete *Bremia lactucae*, several allele balance profiles were described for different isolates. Flow cytometry showed that the total DNA content of nuclei expected to be polyploid was the same as nuclei expected to be diploid. Upon further investigation, it was shown that heterokaryosis and not polyploidy was the cause of the observed variations in allele balance between isolates [4]. Generating allele balance histograms require adequate genome coverage to provide an unambiguous allele balance profile.

Sequencing to high depth (> 50×) for adequate resolution is costly and a barrier to inferences using allele balance, especially when the genome is large. Analysis of individual SNPs in low-coverage data, results in an allele balance profile that cannot resolve the number of haplotypes in an individual. It would be beneficial to develop an objective approach that can classify low coverage data as inconsistent/consistent with diploidy. Such an approach would enable high-throughput, sequence-based screening of populations of ploidy-variable species, such as *Arabidopsis arenosa, Saccharomyces cerevisiae,* and *P. infestans* as well as heterokaryosis in pathogens, such as *B. lactucae*. Previously, nQuire, a Gaussian Mixture Model was developed to classify ploidy directly from read alignments to a reference genome. This approach classified individuals as “diploid”, “triploid”, or “tetraploid” and was validated on downsampled data of *S. cerevisiae* and *P. infestans* [21]. Comparisons of Δ log-likelihoods of the tested model to the free model demonstrated that nQuire could distinguish diploids from polyploids at ~ 20× for *S. cerevisiae*, but more than 20× coverage was required to assign the *S. cerevisiae* isolates to the correct polyploidy (triploid/tetraploid). For *P. infestans* a higher read depth was required to determine the ploidy of the isolates tested [21]. Another approach that summarizes allele balance across multiple windows of a genome assembly was recently deployed in vcfR and was deployed on low coverage data (~ 12×), using allele balance to infer copy number variation of *P. infestans* isolates [5, 23]*.* The latter method aims to determine if parts of the genome are present in higher quantities than expected and reports the proportions of inferred diploidy and polyploidy in each sample. This method requires subjective, specialist interpretation of its results.

The number of haplotypes in an individual will impact the sample variance of allele balance:

$${\text{s}}^{{2}} = \frac{{\mathop \sum \nolimits_{i = 1}^{N} \left( {X_{i} - \overline{X}} \right)^{2} }}{N - 1};{\text{ i}} = {1},{ 2},{ 3}, \, \ldots ,{\text{ N}};{\text{ X}} = {\text{proportion }}\;{\text{of}}\;{\text{ alternate }}\;{\text{allele}},{\text{ X}} \in (0,1).$$

This is because in a diploid, the allele frequency of most SNPs will be 0.5/0.5, resulting in lower variance of the dataset. In a polyploid, many SNPs will not have an allele balance of 0.5/0.5, therefore increasing the variance in the dataset. Variance of allele balance has not previously been reported as an objective approach to discriminate between polyploids from diploids. In this paper, we establish a protocol for utilizing the variance of allele balance to detect departures from the diploid state. This protocol was validated on simulated, synthesized, and downsampled genuine high-coverage whole-genome sequencing data, from a plant, a fungus, and two oomycetes. The protocol was deployed to analyze 21 European race type isolates of *B. lactucae,* demonstrating that 12 were homokaryotic; the other nine were likely heterokaryotic. This protocol allows the use of existing low coverage datasets and generation of additional informative datasets at low cost.

## Results

**Fig. 1:**
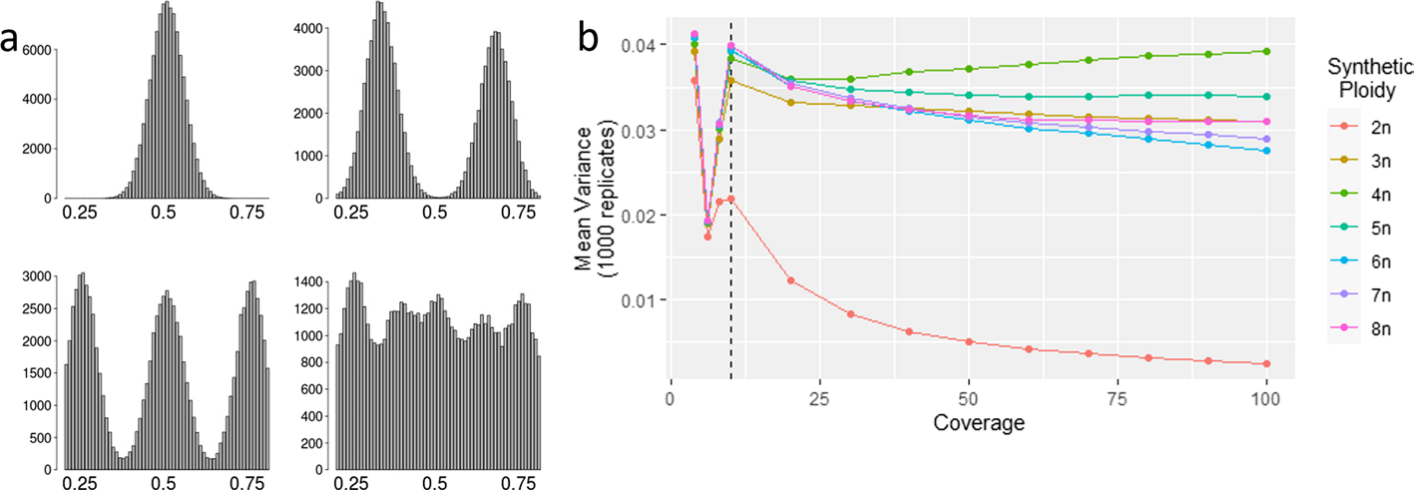
Statistical simulation of variance of allele balance at different ploidy levels. **a** Allele balance histograms of 100,000 loci derived from 2n, 3n, 4n, and 8n datasets simulated to 100× whole-genome coverage. Downsampled allele balance histograms are presented in Additional file 1: Fig S1. **b** Variance of allele balance calculated for 2n to 8n ploidies, with coverages at 4×, 6×, 8×, and 10× to 100× in increments of 10. The simulated diploid can be discriminated from simulated polyploids at coverages over 10× (dashed line). Below 10×, variance of allele frequency begins to converge for all ploidies

The simulation test demonstrated that the variance of allele balance can discriminate between diploids and polyploids. Plotting histograms of simulated data recreated the expected allele frequency plots for ploidies 2n to 8n, provided over 50× coverage was simulated per site (Fig. 1a, Additional file 1: Fig. S1). As the coverage per locus declined, the resolution of the allele frequency plots decreased, although it was still possible to discriminate between diploids and polyploids in this model dataset at coverage as low as 10× (Additional file 1: Fig. S1). Calculating the variance of allele balance demonstrated that there was a large difference between diploids and polyploids at all coverage levels ≥ 10× (Fig. 1b). At 10×, the mean variance of allele frequencies for simulated diploids (2n) was 0.022 (± 8.46 × 10^–05^, 1000 replicates), while simulated polyploids (3n to 8n) had variance of allele frequencies ranging from 0.035 to 0.40 (± 1.2 × 10^–04^, 1000 replicates). As the coverage increased, the variance of allele balance for the simulated diploids declined; for simulated polyploids, the variance of allele balance remained constant (Fig. 1b, Additional file 2: Table S1). The standard deviation of 1000 replicates demonstrated that it was not possible to reliably decipher between different polyploids (> 2n; Additional file 2: Table S1). Therefore, the difference between the variance of allele balance for diploids vs. polyploids means this metric can be used to discriminate diploids from polyploids.

**Fig. 2:**
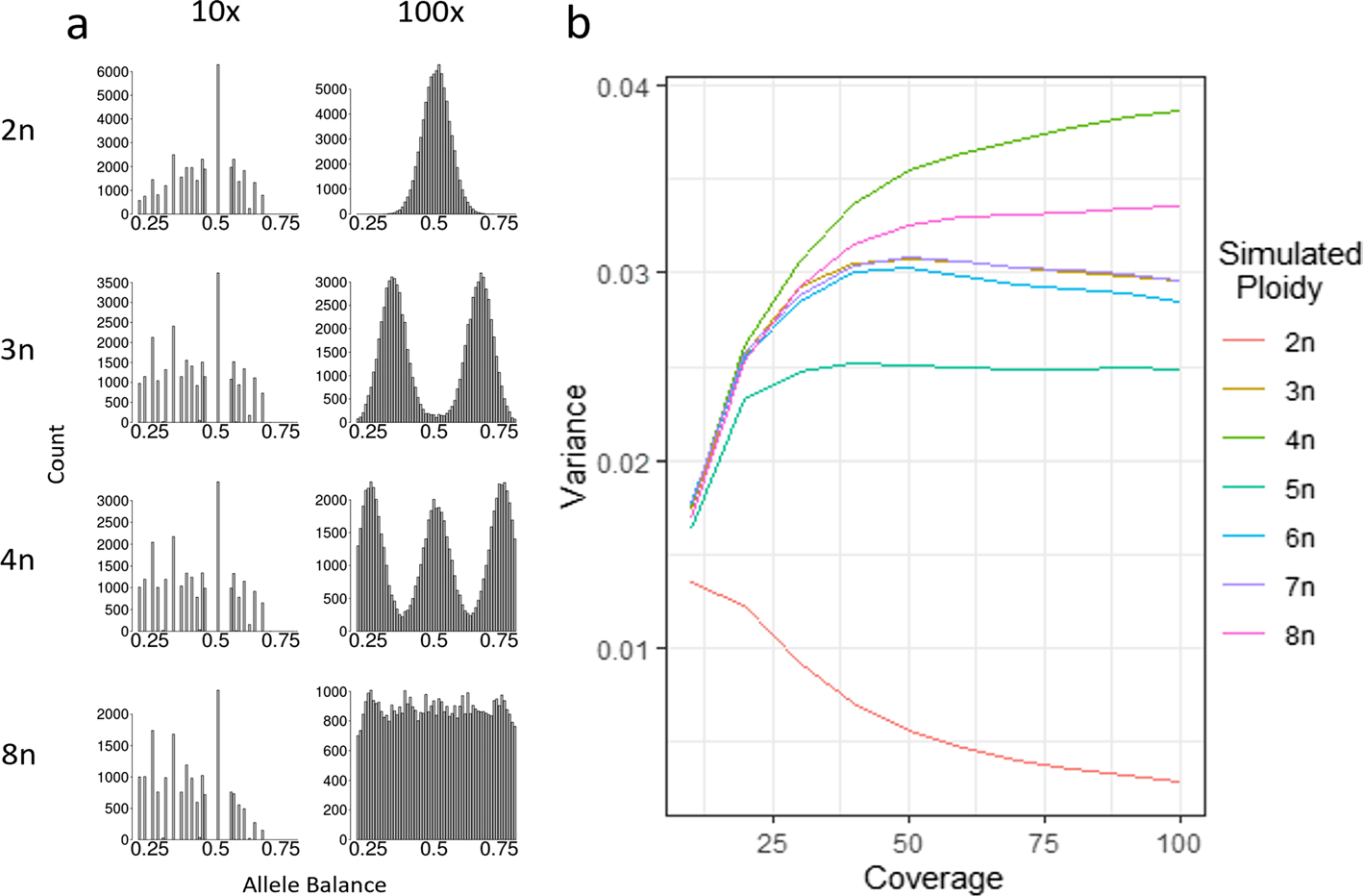
Variance of allele balance derived from reads generated for synthetic *E. coli* “polyploids”. **a** Allele balance histograms produced from variant calls of synthetic *E. coli* datasets using simulated reads. At 10× it is hard to visually distinguish between diploids and polyploids due to the dominant 0.5× bar. At 100× it is possible to visually distinguish between ploidies. Additional downsampled allele balance histograms and ploidies are presented in Additional file 1: Fig S2. **b** Variance of allele frequencies were calculated from the same data and could distinguish the synthetic diploid from synthetic polyploids. The variance of allele frequencies begins to converge at 10×, although they can still be differentiated

Variance of allele balance was able to discern synthetic diploids from polyploids, generated from the *E. coli* genome sequence. Synthetic reads were analyzed with conventional read alignment and variant calling tools (see Materials and Methods). Allele balance plots of SNPs called from reads generated from synthetic diploids and polyploids were able to reproduce expected allele frequency plots at 100× for synthetic ploidies up to and including 8n (Fig. 2a, Additional file 1: Fig. S2). Synthetic diploids could be discerned from synthetic polyploids at lower coverages, but resolution was reduced below 50×. For read sets with five or more synthetic genotypes (≥ 5n), 50× coverage was not adequate to resolve individual peaks in the allele balance histograms, but plots produced were still inconsistent with the expectations of diploidy. At 10× coverage, it was not possible to visually discern diploids from polyploids by comparing the allele balance histograms (Fig. 2a, Additional file 1: Fig. S2). When the variance of allele balance for these same SNP calls was calculated, diploids could be differentiated from polyploids at all coverages from 10× to 100× (Fig. 2b). For synthetic polyploids, variance of allele balance dropped at lower coverages; this was not observed in statistical simulations (Figs. 1b, 2b). This reduced variance of allele balance could be due to the read alignment or variant calling stages. However, the variance of allele balance at 10× coverage was able to discriminate between the synthetic diploid and polyploid individuals using software commonly used for read alignment (BWA) and variant calling (FreeBayes).

**Fig. 3:**
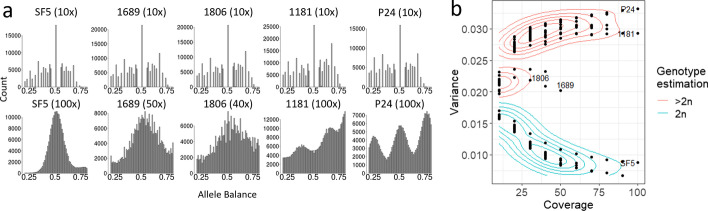
Variance of allele balance calculated for isolates of *B. lactucae* previously confirmed as homokaryotic or heterokaryotic. Whole-genome sequencing datasets of thirty isolates that had been sequenced to high coverage were downsampled to 10× to the highest coverage of factor by tens up to 100×. **a** Allele frequency plots of isolates previously identified as homokaryotic (SF5) or heterokaryotic (all others). At 10× coverage it is not possible to distinguish the homokaryotic isolate from the heterokaryons. At higher coverage, multiple allele peaks are evident corresponding to different numbers of genotypes. **b** Variance of allele balance was calculated from the same downsampled datasets. At 10×, homokaryotic and heterokaryotic isolates could be differentiated from one another. The difference in variance between the two groups increased as sequencing coverage increased. Smoothing through kernel density estimation was used to identify contours around the datasets. Labels indicate isolates presented in A. See the Results section for discussion of isolates 1689 and 1806 that appear different compared to other heterokaryotic isolates, but do not fit the expectations for diploidy at any coverage

When applied to real data, variance of allele balance was able to discriminate between homokaryotic and heterokaryotic isolates of *B. lactucae*. More SNPs were called in heterokaryotic isolates than homokaryotic isolates (Additional file 1: Fig. S3). At high coverage, allele balance histograms were able to discern homokaryotic isolates, such as SF5, from heterokaryotic isolates, including 1689, 1806, 1181, and P24 (Fig. 3a). This is because homokaryotic isolates contain two genotypes, while heterokaryotic isolates contain four or more genotypes. When downsampled to lower coverages, it was not possible to distinguish homokaryons from heterokaryons using allele balance histograms (Fig. 3a, Additional file 1: Fig. S3). In contrast, at all coverage levels, the variance of allele balance was lower for the 12 homokaryotic isolates than for the 18 heterokaryotic isolates (p < 2 × 10 ^−16^). Sampling these isolates at multiple coverages produced curves similar to that of the *E. coli* simulated data (Fig. 2b, 3b). Smoothing around the downsampled *B. lactucae* data produced contours which can be used to classify read sets as homokaryotic or heterokaryotic. The isolates 1689 and 1806 displayed allele balance variances, which, while inconsistent with diploidy, did not fall within the contours established here for heterokaryotic isolates. The allele balance histograms showed that these isolates have unresolved profiles as previously reported [4]. The conclusion from that study was that these isolates are complex heterokaryons, possibly caused by an uneven mixture of multiple genotypes. Consequently, with 10× or higher coverage, a sample is from a heterokaryotic isolate if the variance of allele balance is greater than 0.019. A variance of allele balance lower than 0.019 at sequencing coverages of 10× or higher indicates a homokaryon. Therefore, VCFvariance.pl is able to recapitulate previous conclusions [4] based on lower sequencing coverage.

**Fig. 4:**
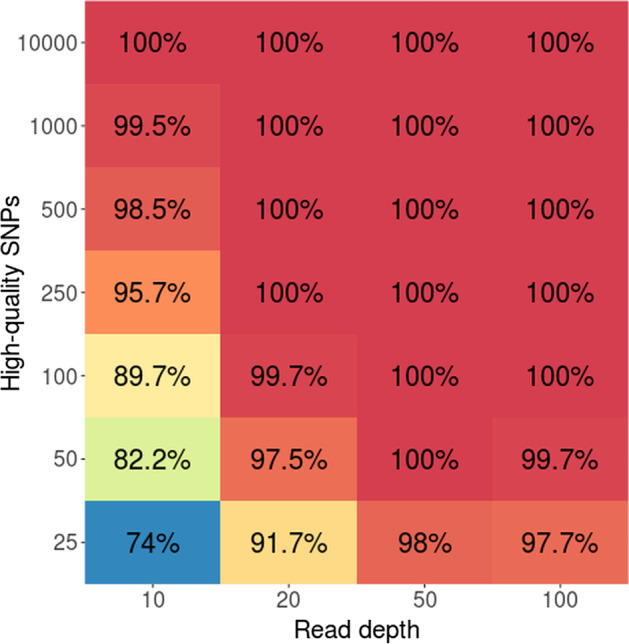
Evaluation of the effect of SNP number on correct assignment. The heatmap indicates the percentage of true positives called for six isolates of *B. lactucae* (three homokaryons, three heterokaryons) with 100 bootstraps of each combination of SNP number and read depth

The number of SNPs required for accurate assessment was investigated by downsampling allele balance calculated for high-quality SNPs called from *B. lactucae*. For 100 bootstrap samples of three homokaryotic isolates (SF5, 1485, and 1486) and three heterokaryotic isolates (1181, P24, and 622b), the accuracy was 100% at all coverages tested (10×, 20×, 50×, and 100×) provided ten-thousand SNPs were analyzed, which is typically far lower than the number of SNPs available (Additional files 5 to 8). At 10× coverage, the accuracy dropped as the number of SNPs analyzed reduced (Fig. 4), reaching 74% when only 25 SNPs were analyzed, which is an unrealistically low number for a shotgun sequencing dataset. The drop in accuracy was not as great at higher coverages; 25 SNPs resulted in an accuracy of 91.7% at 20× coverage and 98% at 50× (Fig. 4). Therefore, variance of allele balance can infer departures from diploidy at high accuracy with fewer SNPs than are likely to be available.

The same six downsampled *B. lactucae* isolates were used to compare VCFvariance.pl to nQuire [21], to determine their relative accuracy at low coverage. For nQuire, homokaryotic isolates were expected to have a lower Δ Log-Likelihood to the diploid model than to the triploid or tetraploid model. This was the case for three isolates previously described as homokaryotic (SF5, 1485, and 1486) at coverages of 20× and above. At 10×, the Δ Log-Likelihood was lower in the triploid model than the diploid model for the homokaryons SF5 and 1486 and would therefore result in false calls (Additional file 1: Fig S4). Heterokaryotic isolates were expected to have a lower Δ Log-Likelihood for the triploid or tetraploid model than the diploid model. This was the case for three isolates previously described as heterokaryotic (1181, 622b, and P24) at all coverages. Therefore, in this analysis, nQuire was suitable to determine if isolates of *B. lactucae* are heterokaryotic or homokaryotic at 20× coverage and above. In contrast, the variance of allele balance for these isolates was able to assign them as heterokaryotic or homokaryotic at 10× coverage and above (Fig. 3).

**Fig. 5:**
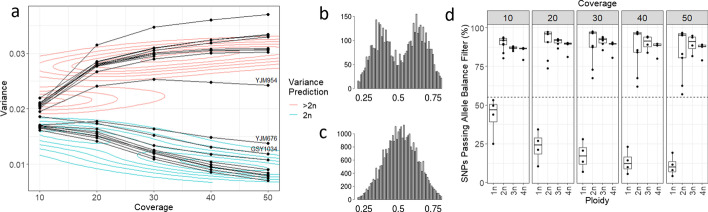
Variance of allele balance calculated for downsampled *S. cerevisiae* isolates exhibiting ploidy variation. Whole-genome sequencing datasets of isolates were downsampled to 10×, 20×, 30×, 40×, and 50×. **a** Variance of allele balance was calculated from downsampled variant calls. Observations calculated from the same isolate are joined by a line. The contours provided as a guide were calculated using *B. lactucae* data (Fig. 3). Histograms of allele balance were investigated for the two labelled isolates. **b** Isolate YJM954 had bimodal distribution of allele balance frequencies inconsistent with diploidy. **c** Isolate YJM676 had a Gaussian distribution consistent with diploidy. **d** The percentage of high-quality SNPs called as heterozygous could differentiate haploid isolates from polyploidy isolates. At all coverages the percentage of SNPs passing the allele balance filter never exceeded 55% for haploid isolates and was never less than 55% for diploid or polyploid isolates. The closest points were 53.1% for haploid isolate CBS1227 calculated from 135 polymorphisms at 10× and 57.1% for diploid isolate CBS2910 calculated from 2377 SNPs at 50×. See Additional file 2: Fig S5 for more details

The protocol was further validated by downsampling high coverage sequencing data of 24 isolates of *S. cerevisiae.* This analysis surveyed nine diploids and eleven polyploids as well as four haploids. As expected, the variance of allele balance was higher for polyploids than for diploids at all coverages tested (p < 2 × 10^–16^, Fig. 5a). When downsampled to 10×, the variance of allele balance for isolate GSY1034 provided weak support for diploidy because it was on the edge of the contours defined by *B. lactucae* (variance = 0.01855). At higher coverage, GSY1034 was clearly diploid. This demonstrates potential ambiguity at low coverage that may require additional sequencing to resolve. Histograms of allele balance were investigated for two high coverage datasets that, at high coverage, fell outside of the contours defined by *B. lactucae*. For isolate YJM954, the frequency of allele balance was bimodal, inconsistent with diploidy (Fig. 5b). For YJM676, the frequency of allele balance was consistent with diploidy (Fig. 5c). These isolates were assigned to the correct ploidy using the 0.019 cutoff established using *B. lactucae* data. Across all isolates, more SNPs were called and passed the allele balance filter for polyploid isolates than diploid isolates (Additional file 1:Fig. S5), as found for *B. lactucae*. Running the four haploid isolates of *S. cerevisiae* through VCFvariance.pl demonstrated that haploids could be identified, because polymorphisms called versus the reference assembly should not be heterozygous in haploid isolates. For haploid isolates at 50×, between 161 to 190 SNPs were identified, of which only 4.2% to 19.3% passed the allele balance filter (see “Methods”); in comparison, at least 57% of the SNPs passed the allele balance filter at 50× coverage of diploids and polyploids. At 10×, between 135 and 231 SNPs were identified in haploids, of which 25% to 53.1% passed the allele balance filter; in contrast, at least 79% of the SNPs passed the allele balance filter at 10× coverage in diploids and polyploids. Consequently, the percentage of polymorphisms passing the allele balance filter could be used to distinguish haploids (Fig. 5d, Additional file 1: Fig S5). Therefore, variance of allele balance can be used to discriminate between haploids, diploids, and polyploids in isolates of *S. cerevisae*.

**Fig. 6:**
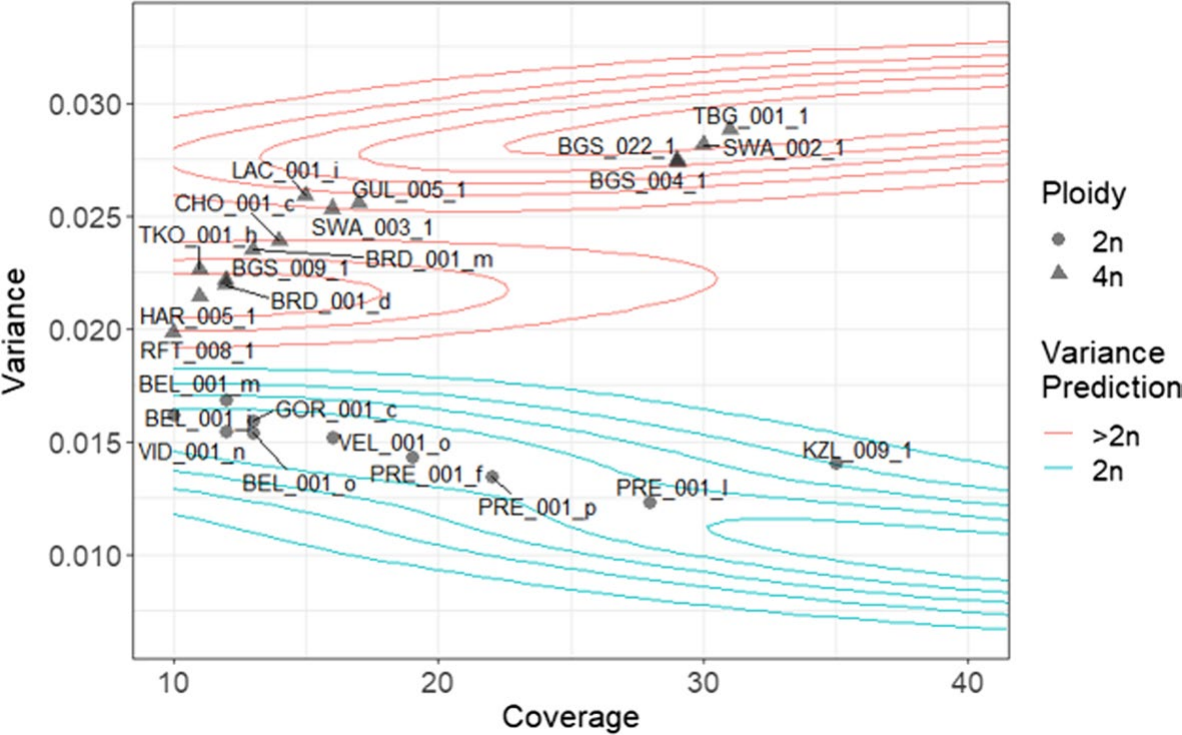
Variance of allele balance calculated for 24 *A. arenosa* individuals. Individuals previously described as diploid (circles) had a lower variance of allele balance compared to polyploid (triangles) individuals. Variance values calculated for all individuals fell within the contours defined for *B. lactucae* (Fig. 3)

Variance of allele balance was investigated for populations of *A. arenosa* that had been previously established as diploid or polyploid (Fig. 6). The sequencing coverage of samples surveyed ranged from 10× to 35×. As with previous observations, more SNPs were identified for polyploids than for diploids and more SNPs were identified in individuals sequenced to higher coverage (Additional file 1: Fig S6). The variance of allele balance was higher for polyploids than for diploids (p = 3.16 × 10^–10^) and fell within the contours defined by heterokaryotic and homokaryotic isolates of *B. lactucae*. The classification of 24 individuals as diploid/polyploid was 100% correct. Therefore, variance of allele balance calculated for *A. arenosa* was able to discriminate between populations previously established to be diploid or polyploid.

**Fig. 7:**
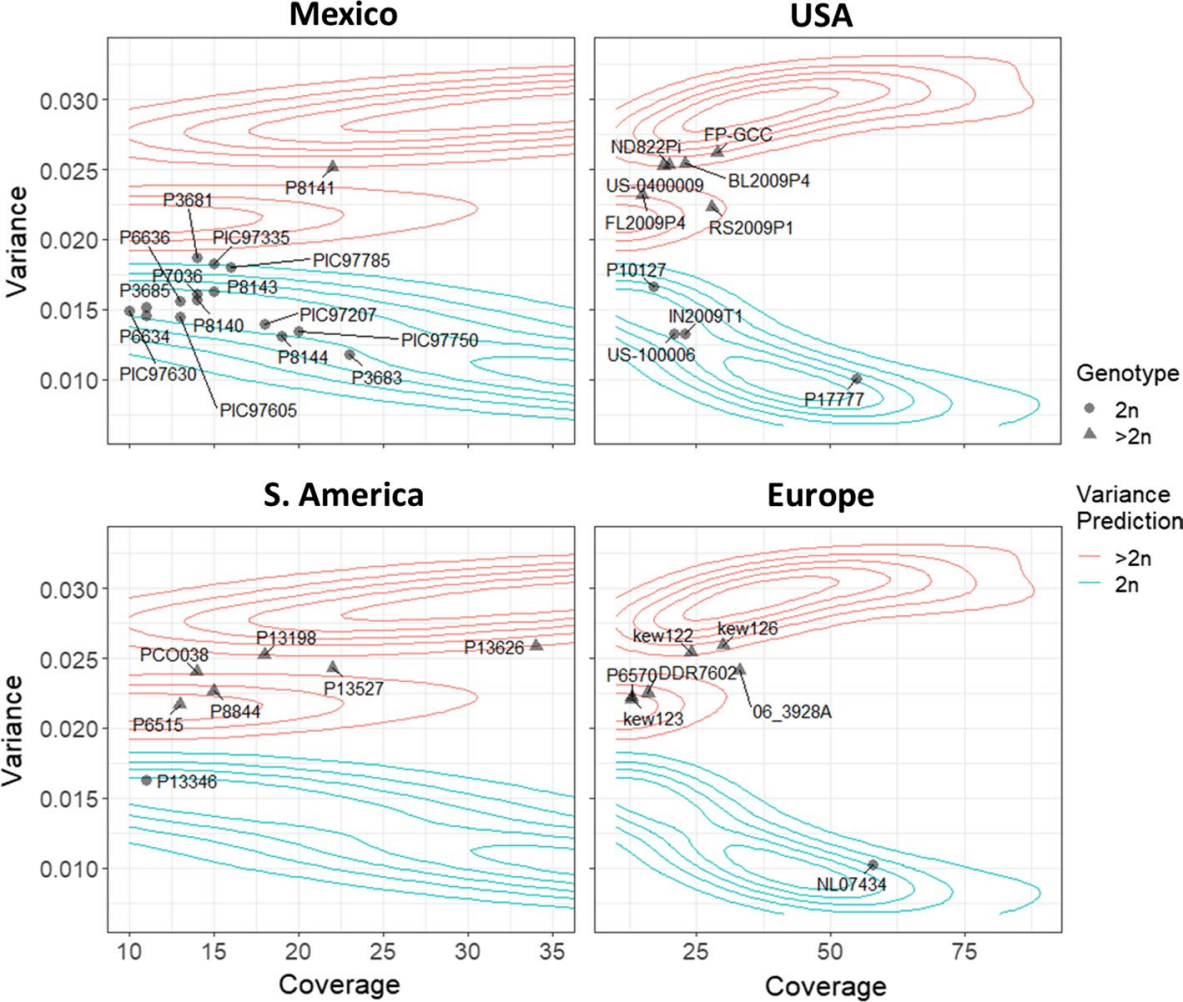
Variance of allele balance calculated for 40 isolates of *P. infestans*. Panels represent the geographic location the isolate was collected from as reported in other studies [5, 25]. Isolates previously described inconsistent with diploidy (triangles) had higher variance of allele balance than isolates consistent with diploidy (circles). Variance values calculated for most isolates fell within the contours defined for *B. lactucae* (Fig. 3)

Variance of allele balance analysis for 40 *P. infestans* isolates identified 21 as diploid; 19 were inconsistent with diploidy (Fig. 7). Most isolates fall within the contours defined by *B. lactucae*. Consideration of the variance plots by geographic region demonstrated that all but one of the 16 isolates analyzed from Mexico were diploid, consistent with sexual reproduction occurring in the region. Four of the ten isolates surveyed from the USA, one of the seven from South America, and one of the six isolates surveyed from Europe were diploid. This panel included three herbarium specimens collected in the 1950s, labeled kew122, kew123, and kew126, that were all inconsistent with diploidy. These results are 100% consistent with the previously reported polyploidy and copy number variation described for these isolates [5, 12, 13]. The variance of allele balance was significantly higher for isolates inconsistent with diploidy (p < 2 × 10^–16^).

**Fig. 8:**
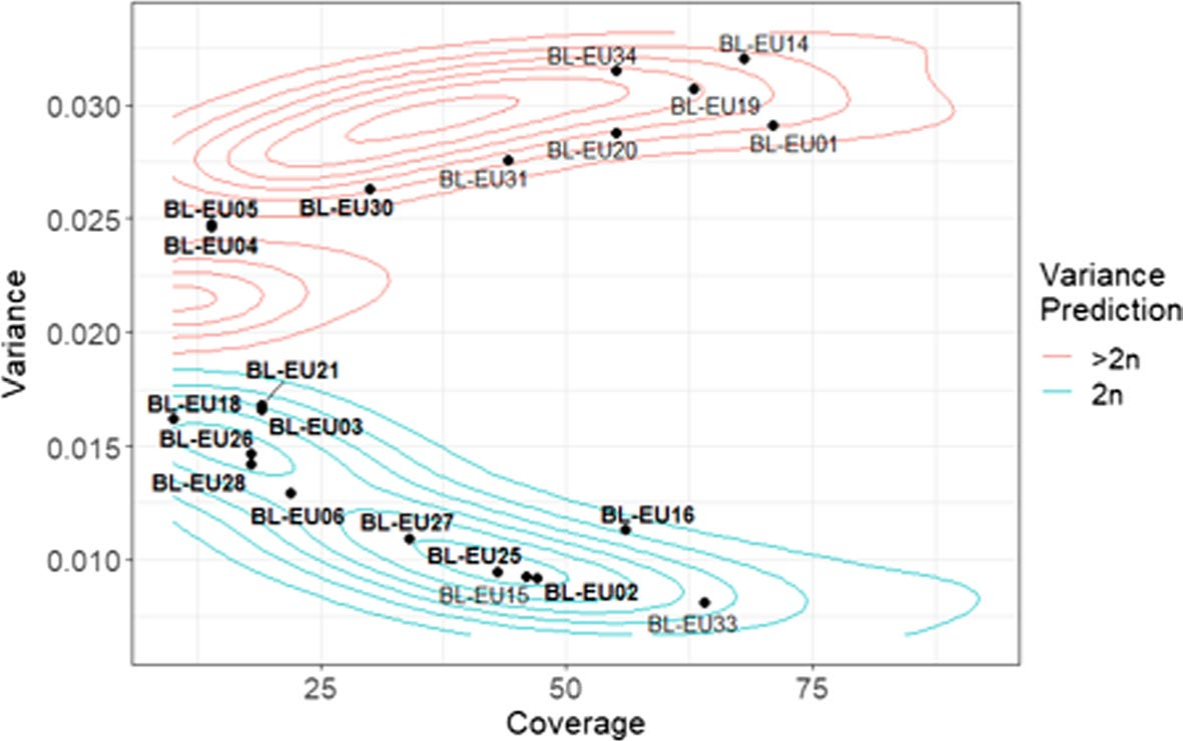
Variance of allele balance calculated for 21 European race type isolates of *B. lactucae*. Contours calculated in Fig. 3 indicated that 12 isolates were homokaryotic. The other nine isolates are likely heterokaryotic and fall within contours calculated for heterokaryons. Bold-faced labels indicate 13 isolates for which the heterokaryotic condition was not previously known. Plain-faced labels indicate isolates for which histograms of allele balance have been reported [4]; these isolates were used to generate Fig. 3.

Finally, VCFvariance.pl was deployed to investigate the nuclear state of type isolates for European races of *B. lactucae*. Sequences were generated for 13 isolates and variance of allele balance was calculated for these and eight previously reported isolates [4] sequenced to high coverage and used to validate the protocol (Additional file 2: Table S2 and S6). Twelve of the 21 isolates were diploid. The remaining nine, BL-EU01, BL-EU04, BL-EU05, BL-EU14, BL-EU19, BL-EU20, BL-EU30, BL-EU31, and BL-EU34, were inconsistent with diploidy and may therefore be heterokaryotic (Fig. 8). The heterokaryotic nature of these isolates could result in unstable virulence phenotypes due to fluctuations in the populations of their nuclei [4].

## Discussion

We show that the variance of allele balance is an effective measure to distinguish a sample with two genotypes (diploid, homokaryotic), from those with more than two genotypes (polyploid, heterokaryotic, or mixed culture). The reliability of this approach was demonstrated using simulated (Fig. 1), synthesized (Fig. 2), and authentic sequencing data (Figs. 3, 4, 5, 6). This approach is robust and can be applied in multiple biological situations. In species with variable ploidy levels, such as *A. arenosa* [24] and *S. cerevisae* [8], the variance of allele balance was consistently higher for polyploids compared to diploids at all coverages tested over or equal to 10×. For *B. lactucae*, it correctly differentiated previously characterized heterokaryotic isolates from homokaryotic isolates [4] at all coverages tested and determined whether 13 race type isolates were homokaryotic. For *P. infestans*, it classified isolates as consistent or inconsistent with diploidy as previously established [5]. This approach was also successfully applied to herbarium samples of *P. infestans* [5, 25]. The variance of allele balance reflects the number of haplotypes present regardless of whether it originates from a plant, animal, or microbe. This meant that the same kernel density estimate contours, calculated by downsampling *B. lactucae*, could be plotted as a visual guide across all datasets (Figs. 3–7). As a general rule, using default conditions (40% deviation on coverage as specified by -d), diploids/homokaryons can be confidently assigned at 10× if the variance is below 0.019; polyploids/heterokaryons if the variance is above 0.019; however, results around 0.019 may require further sequencing depth for clarification. This boundary can be applied at higher coverages, but it is expected that the variance of allele balance for diploids/homokaryons will be even lower and vice versa for polyploids/heterokaryons. Users should be aware that changing the standard deviation of coverage (specified by -d) will affect the variance values obtained. In summary, calculating the variance of allele balance is a robust method for detecting deviations from the diploid state.

This strategy is successful because an individual with more than two genotypes will have allele frequencies that significantly deviate from the expected 0.5/0.5 ratio [4, 13]. Calculating variance of allele balance enables interpretation at much lower coverages than the 50× used previously and can therefore reduce the cost of detecting polyploids and heterokaryons by up to 80% (sequencing to 10× rather than 50×). The shortcomings of plotting variance of allele balance are the same as plotting histograms of allele balance: such analyses may lead to false conclusions if there has been recent hybridization between two highly homozygous species or recent whole-genome duplication. In these instances, the expected allele balance of a tetraploid would be predominantly 1:1, despite there being four copies per locus. Variance of allele balance cannot delineate the number of genotypes in polyploids or heterokaryons at any coverage; manual inspection of allele balance plots generated from high coverage data retain value for determining the number of genotypes in a polyploid or heterokaryotic individual. Finally, at low coverages there may be ambiguity in the results because the variance of allele balance for diploids and polyploids begin to converge, as shown with downsampled simulated and authentic data (Figs. 2, 3, 4). Data points that fall outside of the contours at low coverage may require higher coverage to conclude whether they are consistent with diploidy. Therefore, plotting the coverage on the x-axis conveys the confidence in the call made by VCFvariance.pl. An example of such a point can be seen in the *S. cerevisae* data, where at 10× the isolate GSY1034 borders the diploid call, but at higher coverages is confidently called to be diploid (Fig. 4).

Calculating the variance of allele balance allows efficient and objective presentation of data while avoiding potential ambiguity. Presentation of allele balance histograms requires one plot per sample [4, 5]; in contrast, communication of results from the variance of allele balance for multiple samples can be presented in a single plot. Interpretation of allele balance histograms can be subjective and may require experienced interpretation and explanation. Calculating the variance of allele balance provides a single value indicative of whether the genotype of an individual is consistent with diploidy. Therefore, using the variance of allele balance to infer departures from diploidy reduces ambiguity in the interpretation of results. This method provides a computationally light-weight solution that enables whole-genome ploidy analyses at low sequencing coverage.

## Conclusions

This study demonstrated a methodology for reliable detection of the departure from a diploid state using low coverage whole-genome sequencing data of any eukaryotic species. Such departures may be due to polyploidy, heterokaryosis, a mixed sample, or chromosomal copy number variation. This protocol can also detect haploid samples. Deployment of this strategy is computationally inexpensive and can reduce the sequencing costs by up to 80%. This protocol has been used to determine the heterokaryotic status of type isolates of *B. lactucae* and can be readily deployed to test for departures from diploidy in any organism.

## Methods

### Protocol validation

Simulated datasets were generated to capture allele frequencies for 100,000 loci with ploidy levels ranging from 2 to 8n at coverages ranging from 10× to 100× using rbinom() in R [26]. Allele frequency profiles were filtered to only consider frequencies between 0.2 and 0.8, a common criterion to filter for heterozygous variants [4, 12, 13]. Results were rounded to two decimal places, tabulated, and plotted using the base barplot(). Variance for each simulation was calculated with var() and plotted as a line graph using ggplot2 [27]. Variance calculations were replicated 1000 times using repeat() to obtain mean and standard deviation statistics.

Diploid and polyploid genotypes were synthesized in silico from *Escherichia coli* strain K-12 (GCA_000005845.2) by introducing mutations into the reference assembly and proliferating them to the desired ploidy level. The number of polymorphisms at each ploidy level are detailed in Additional file 2: Table S7. Synthetic genotypes were generated using BedTools v2.25.0 maskfasta [28], masking with the nucleotide to be mutated. Synthetic genotype sequences were combined with the original assembly to generate templates for read generation. Synthetic 150 bp reads were generated from these combined assemblies using randomreads.sh [29], generating 100× coverage.

Authentic reads of 30 *Bremia lactucae* isolates sequenced to high coverage and previously determined to be homokaryotic or heterokaryotic [4] were downloaded from SRA BioProject PRJNA387192 (Additional file 2: Table S2). Reads of 40 *Phytophthora infestans* isolates sequenced to varying coverages were downloaded from several SRA accessions (Additional file 2: Table S3). These reads have been used previously to infer diploidy, polyploidy, or continuous copy number variation [5, 13]. Reads of 24 *Saccharomyces cerevisiae* isolates that exhibited ploidy variation [8] were downloaded from SRA BioProject PRJNA315044 (Additional file 2: Table S4). Reads of 24 *Arabidopsis arenosa* accessions from 17 European populations, which were characterized as diploid or tetraploid [24], were downloaded from SRA BioProject PRJNA484107 (Additional file 2: Table S5).

Simulation and genuine reads were deduplicated with SuperDeduper [30], then aligned to their respective GenBank reference sequences (*E. coli*; GCA_000005845.2, *B. lactucae*; GCA_004359215.1, *P. infestans*; GCA_000142945.1, *S. cerevisiae*; GCA_000146045.2, and *Arabidopsis arenosa*; GCA_905216605.1) using BWA-MEM v0.7.16 [31]. Variants were called with FreeBayes v1.2 [32]. The genome wide coverage was calculated by BEDtools [28]. A Perl script (VCFvariance.pl) was implemented to calculate the variance of allele balance of SNPs filtered to be within 40% coverage of the genome wide coverage; this can be altered to include fewer or more polymorphisms with the -d flag. Allele balance between 0.2 and 0.8, mapping quality > 30, and genotype quality > 30 was filtered for to eliminate sequencing error. In addition, the Perl script flagged potential haploid accessions (e.g. in *S. cerevisiae*) if less than 55% of the SNPs passing coverage, mapping quality, and genotype quality filters failed the allele balance filter. This final option can be modified using the -p flag if necessary. For *E. coli* (synthetic polyploids), *B. lactucae*, and *S. cerevisiae,* BAM files were downsampled using SAMtools [33], and variant calling and variance calculation were repeated as above. Bar plots were generated with R base barplot() and table() functions [26]. Variance of allele balance were plotted per individual using either geom_line() or geom_point() [27] and labelled with ggrepel() [34]. For *B. lactucae*, kernel density estimation was plotted using geom_density_2d() [27]. Contours produced from the estimation of *B. lactucae* kernel density were also plotted in the *P. infestans, A. arenosa,* and *S. cerevisiae* figures for comparative analysis. For authentic data, linear models were used to determine whether the variance for polyploids/heterokaryons was different compared to diploids/homokaryons

$$Variance \sim Coverage + Ploidy$$

VCFvariance.pl is available at https://github.com/kfletcher88/VCFvariance.

The number of SNPs required for accurate detection for departures from diploidy was investigated by downsampling the arrays of allele balance values calculated from high-quality SNPs. Arrays were queried to generate new arrays of 25, 50, 100, 250, 500, 1000, and 10,000 SNPs and bootstrapped 100 times. The VCFs for homokaryotic isolates SF5, 1485, and 1486, and heterokaryotic isolates 1181, 622b, and P24 calculated from reads downsampled to 10×, 20×, and 50× were used. In addition, VCFs of SF5, 622b, and P24 calculated from 100× coverage were also queried. True positives were called for homokaryons if the variance of allele balance was under 0.019, for heterokaryons if it was over 0.019. Accuracy was calculated as the number of true positives divided by the number of bootstraps and plotted as a heatmap using ggplot2 [27].

VCFcariance.pl was compared to nQuire [21] using the downsampled BAM files of the same set of homokaryotic and heterokaryotic *B. lactucae* isolates used to test the accuracy. Bin files were created using the nQuire subcommand create, specifying a minimum quality of 30. Models of ploidy were assessed using the lrdmodel subcommand. For each isolate, the Δ log-likelihood to each model was plotted using ggplot2 [27]. True positives were called for homokaryons if the Δ Log-Likelihood was lower to the diploid model than either the triploid or tetraploid model. True positives were called for heterokaryons if the Δ log-likelihood was lower to either the triploid or tetraploid model than the diploid model.

### Application of protocol

Low coverage sequencing of European type isolates of *B. lactucae* was used to demonstrate utility of this protocol. Spore pellets in ethanol for type isolates of European races of *B. lactucae* were provided by Diederik Smilde (Naktuinbouw, The Netherlands). DNA was extracted as described previously [4]. Paired-end (~ 300 bp fragments) libraries were prepared using the Kapa HyperPrep kit following the manufacturer’s instructions (Roche, Switzerland). Libraries were sequenced on a MiSeq 2500 or HiSeq 4000. Reads were uploaded to NCBI BioProject PRJNA387192. Reads were deduplicated using SuperDeduper [30], trimmed for adapters and low quality sequence using BBMAP bbduk.sh [29] and mapped with BWA mem v0.7.16 [31]. SNPs were called with FreeBayes v1.2 [32] and the variance of allele balance for each isolate was calculated using VCFvariance.pl (https://github.com/kfletcher88/VCFvariance). One point per isolate was plotted as coverage by variance. Kernel density estimates of established homokaryotic and heterokaryotic *B. lactucae* isolates were plotted as a guide using geom_density2d with six bins [27]. This plot was inspected to determine whether the isolates fit the model of a homokaryotic diploid.

## Supplementary Information

**Additional file 1 Figures S1** Histograms of allele balance generated from simulated data; **Fig. S2** Histograms of allele balance generated from synthetic *E. coli* data, **Fig. S3** Polymorphisms identified for 30 isolates of *B. lactucae*, downsampled to different coverages, used to generate Fig. 3; **Fig. S4** Results of running six isolates of *B. lactucae* through nQuire; **Fig. S5** Polymorphisms identified for 24 isolates of *S. cerevisiae*, downsampled to different coverages, used to generate Fig. 5; and **Fig. S6** Polymorphisms analyzed for 24 *A. arenosa *individuals used to generate Fig. 6 (PDF 1167 kb).

**Additional file 2 Tables S1** Summary results for 1,000 tests simulating different ploidies and different levels of coverage; **Table S2** SRA information and values calculated for downsampled data of *Bremia lactucae *isolates used to validate the variance of allele balance approach; **Table S3** SRA information and values calculated for *Phytophthora infestans* isolates used in this study; **Table S4** SRA information and values calculated for downsampled *Saccharomyces cerevisiae* isolates used in this study; **Table S5** SRA information and values calculated for *Arabidopsis arenosa* individuals used in this study; **Table S6** Variance of allele balance values calculated for European race type isolates of *Bremia lactucae* in this study; and Table S7 Number of mutations introduced into each synthetic haplotype. Lower numbers are a subset of the larger preceding number. Each table is on a separate tab of the excel workbook (XLSX 35 kb).

## Data Availability

New data generated during this project are available under BioProject PRJNA387192. Data downloaded from NCBI for validation of this approach are listed in Additional file 2: Tables S2–S5.
