# Supplementary material for: Variance of allele balance calculated from low coverage sequencing data infers departure from a diploid state
Source: BMC Bioinformatics. 2022 Apr 25;23:150. doi: 10.1186/s12859-022-04685-z (PMC9040317; doi:10.1186/s12859-022-04685-z)
Supplement: Supplementary file 1 — Additional file 1 Figures S1 Histograms of allele balance generated from simulated data; Fig. S2 Histograms of allele balance generated from synthetic E. coli data, Fig. S3 Polymorphisms identified for 30 isolates of B. lactucae, downsampled to different coverages, used to generate Fig. 3; Fig. S4 Results of running six isolates of B. lactucae through nQuire; Fig. S5 Polymorphisms identified for 24 isolates of S. cerevisiae, downsampled to different coverages, used to generate Fig. 5; and Fig. S6 Polymorphisms analyzed for 24 A. arenosa individuals used to generate Fig. 6 (PDF 1167 kb). [file 12859_2022_4685_MOESM1_ESM.pdf]

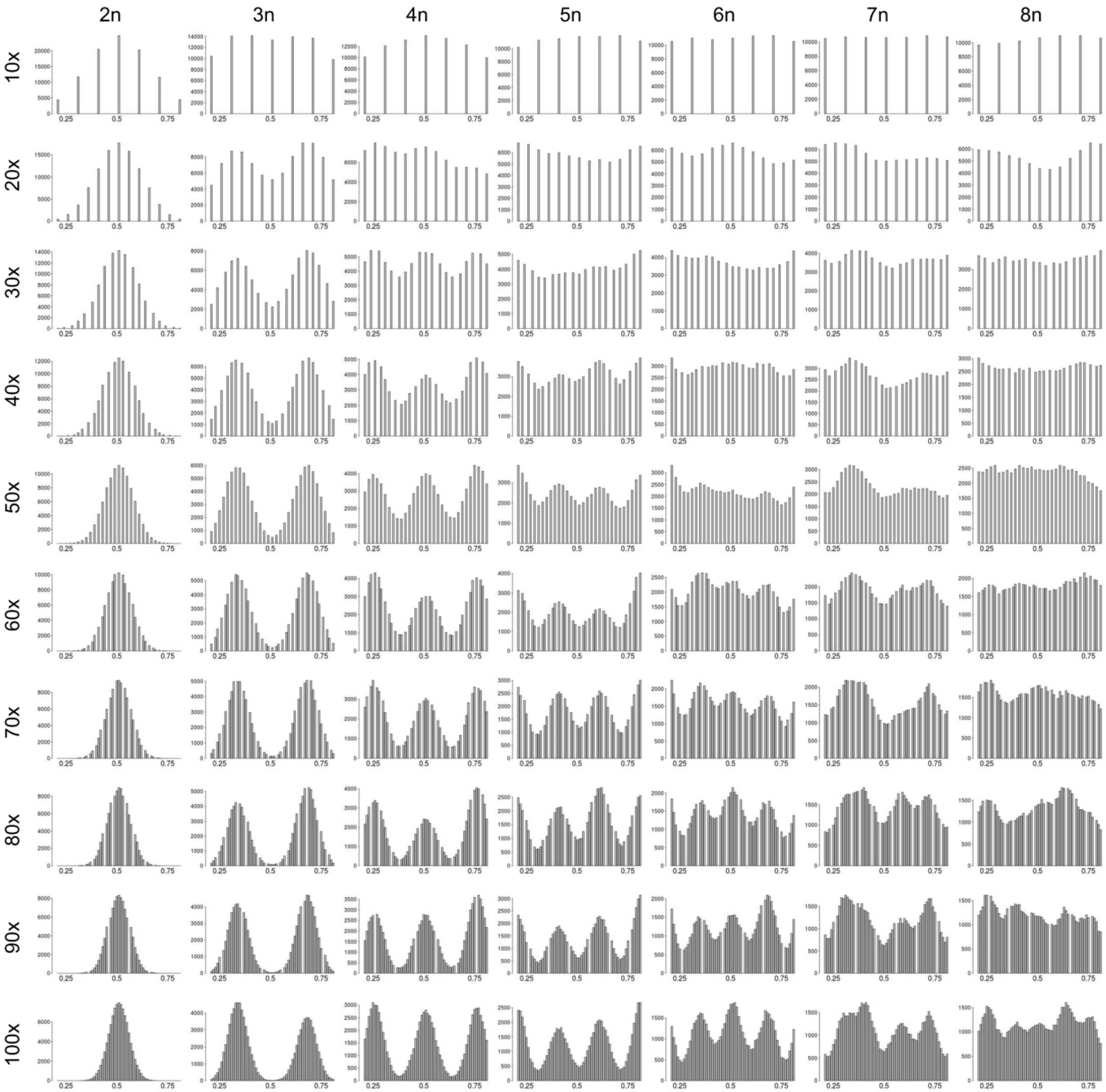

Fig. S1. **Histograms of allele balance generated from simulated data.** The grid indicates expected allele balance for diploids through to octoploids sequenced from 10x to 100x.

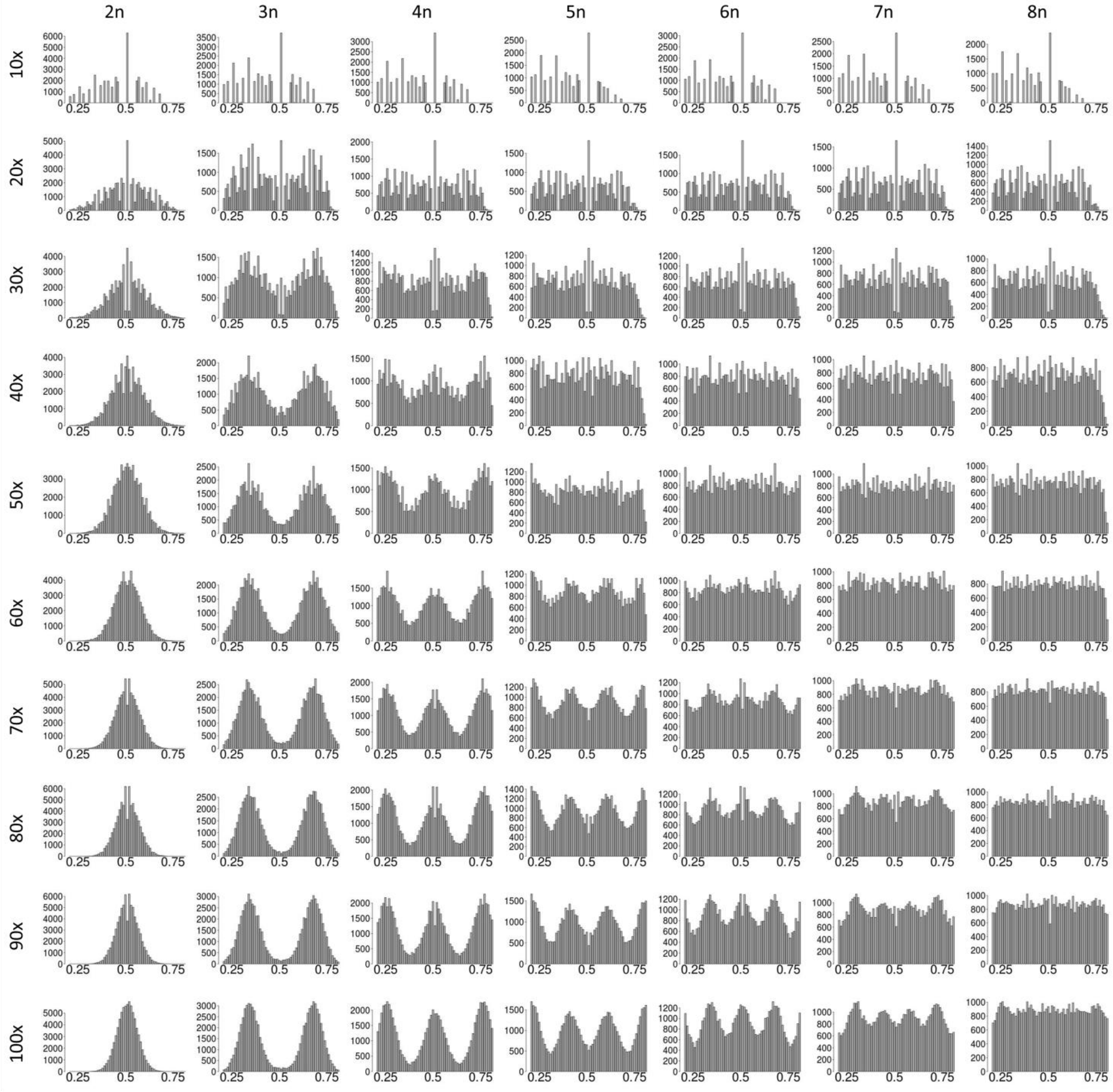

Fig. S2. **Histograms of allele balance generated from synthetic *E. coli* data.** The grid indicates allele balances calculated from diploids through octoploids sequenced from 10x to 100x. Synthetic reads were processed using BWA-mem and Freebayes. In contrast to Figure S1, Supplementary Figure S2 demonstrates the difficulty in subjectively discerning diploids from polyploids at lower coverages.

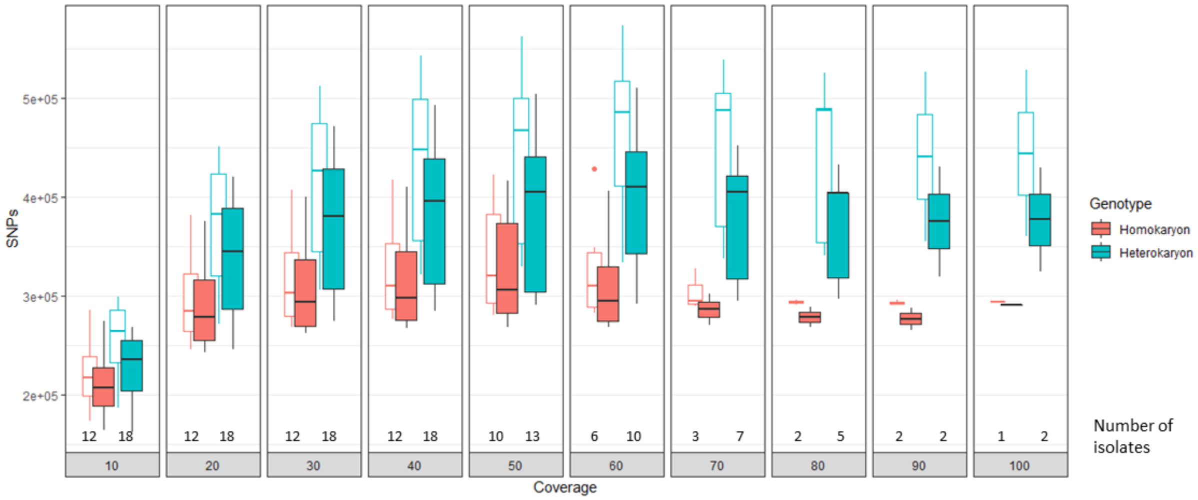

Fig. S3. Polymorphisms identified for 30 isolates of *B. lactucae*, downsampled to different coverages, used to generate Fig. 3. The outlined boxplots indicate the number of high-quality polymorphisms of *B. lactucae* and filled boxplots indicate the number polymorphisms that passed the allele balance filter (i.e., were heterozygous). The number of polymorphisms called for all isolates increased with coverage up to 50x. Beyond 50x the number of polymorphisms plateaued, indicating data saturation. At all coverages, more polymorphisms were identified for heterokaryons than homokaryons. A higher percentage of the polymorphisms passed the allele balance filter for homokaryons than heterokaryons, indicated by the red filled boxplots being closer to the red outlined boxplots.

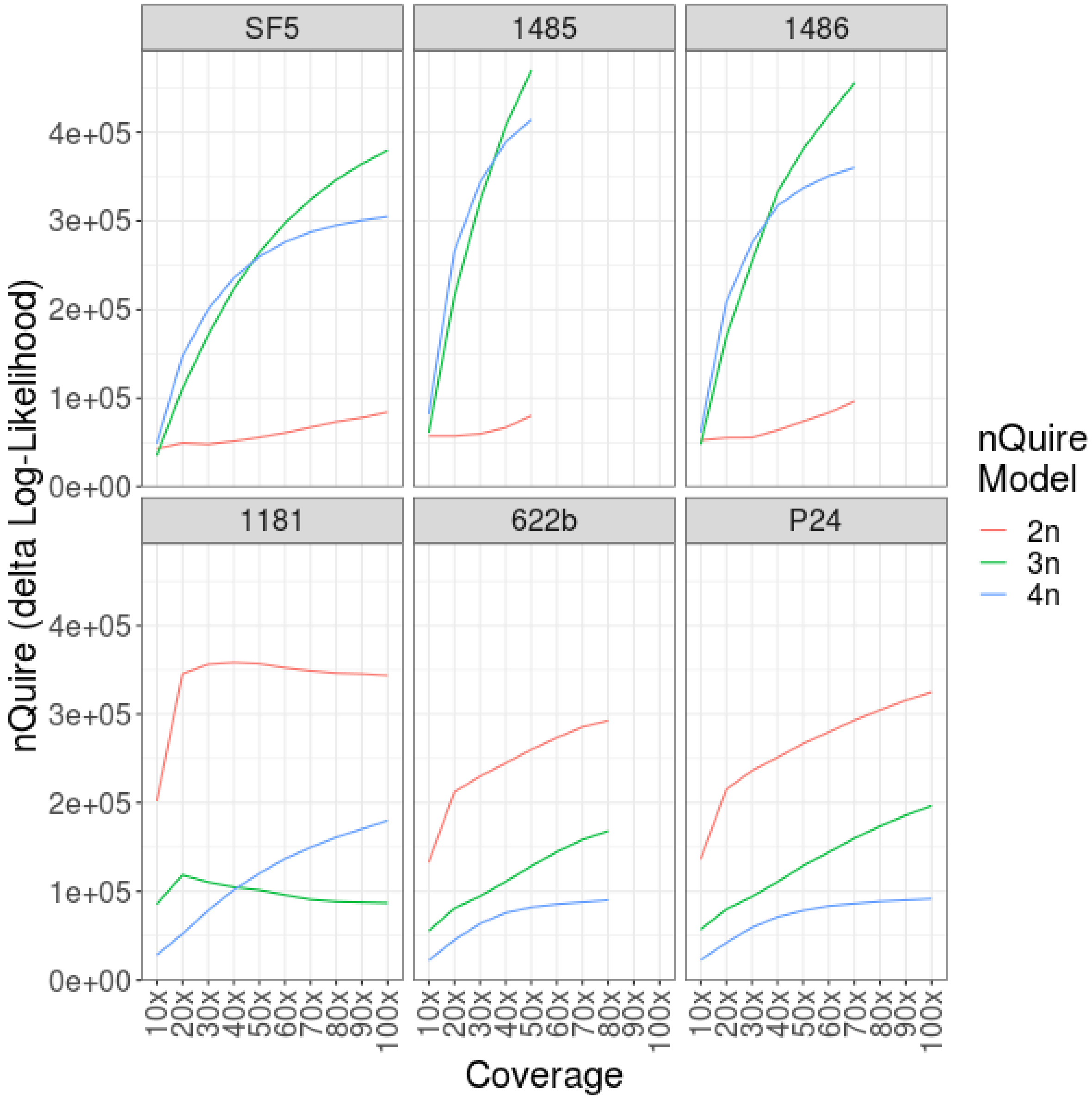

Fig. S4. Results of running six isolates of *B. lactucae* through nQuire. The Δ log-likelihood was lower to the diploid model for homokaryotic isolates sequenced to 20x or higher. At 10x homokaryotic isolates SF5 and 1486 had lower Δ log-likelihood to the triploid model than the diploid model, which would have led to incorrect assignment. At all coverages, heterokaryotic isolates had lower Δ log-likelihoods to the triploid and/or tetraploid model than the diploid model which would have resulted in correct assignment.

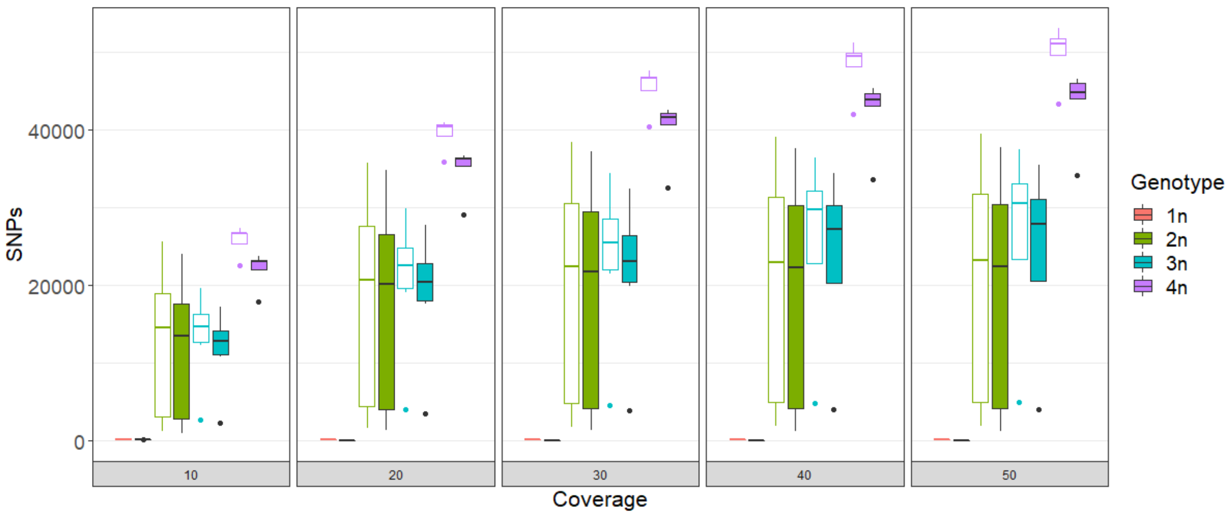

Fig. S5. Polymorphisms identified for 24 isolates of *S. cerevisiae*, downsampled to different coverages, used to generate Fig. 5. The outlined boxplots indicate the number of high-quality polymorphisms of *S. cerevisiae* and filled boxplots indicate the number of polymorphisms that passed the allele balance filter (i.e., were heterozygous). At all coverages, the number of polymorphisms identified for haploids was much lower than for diploids or polyploids. More polymorphisms were identified for tetraploids than triploids and for triploids than diploids. At all coverages, a higher percentage of the polymorphisms passed the allele balance filter for diploids (green) than triploids (turquoise) and for triploids than

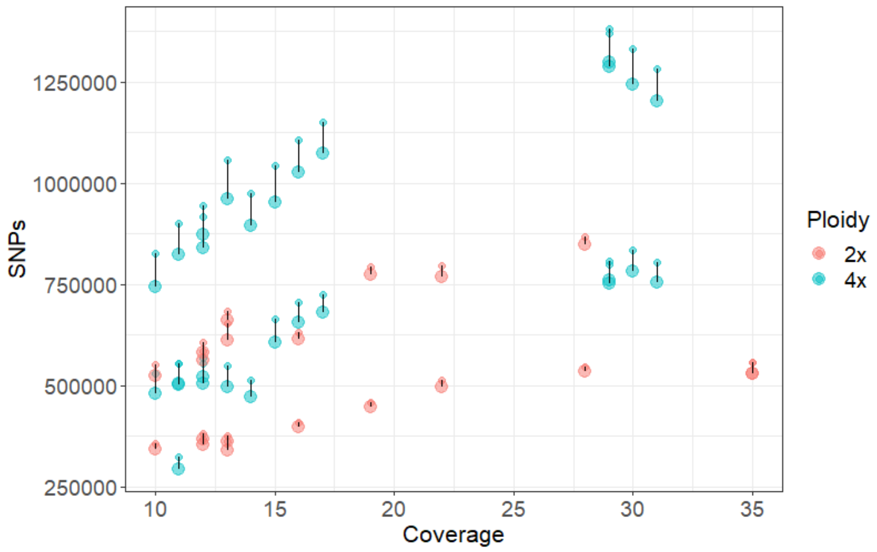

Fig. S6. Polymorphisms analyzed for 24 *A. arenosa* individuals used to generate Fig. 6. The colors indicate whether the individual originates from a diploid or a polyploid population. Smaller circles indicating the number of high-quality polymorphisms are linked by a black line to larger circles, indicating the number of polymorphisms that passed the allele balance filter (i.e., were heterozygous). More polymorphisms were identified for individuals from polyploid populations than from diploid populations. The percentage of variants passing the allele balance filter was higher for individuals from diploid populations than for individuals from polyploid populations.
